# Additive effects of obesity and vitamin D insufficiency on all-cause and cause-specific mortality

**DOI:** 10.3389/fnut.2022.999489

**Published:** 2022-10-21

**Authors:** Shuaihua Song, Yuan Yuan, Xiaolong Wu, Di Zhang, Qianjin Qi, Haoran Wang, Li Feng

**Affiliations:** ^1^Shandong Provincial Hospital Affiliated to Shandong First Medical University, Jinan, Shandong, China; ^2^Department of Clinical Nutrition, Shandong Provincial Hospital Affiliated to Shandong First Medical University, Jinan, Shandong, China; ^3^The First Affiliated Hospital of Shandong First Medical University, Jinan, Shandong, China; ^4^Shandong Provincial Hospital, Cheeloo College of Medicine, Shandong University, Jinan, Shandong, China

**Keywords:** vitamin D, abdominal obesity, obesity, mortality, all-cause, cancer, cardiovascular

## Abstract

Obesity and vitamin D deficiency are both considered risk factors for mortality, but the potential additive effects of vitamin D status and obesity on mortality has not been well-studied. We aimed to examine the possible additive effects of obesity and vitamin D status on all-cause and cause-specific mortality. The data from the NHANES III (1988–1994) and NHANES 2001–2014 surveys were used, and multivariate Cox regression models were performed to assess the additive effects of vitamin D status and overweight/obesity/abdominal obesity on the all-cause, cardiovascular and cancer mortality, by stratifying Cox Hazard Ratios (HRs) across different categories of vitamin D status and body mass index (BMI) and waist circumference (WC) categories. The models were adjusted for age, race/ethnicity, gender, educational level, family income to poverty ratio, leisure-time physical activity, smoking, and drinking. Across all BMI/WC categories, there was an additive effect of the vitamin D both insufficiency and deficiency on all mortality rates, with deficiency having much stronger effect than insufficiency. Interestingly, the effect of vitamin D deficiency overcame the effect of obesity on all mortality rates. The highest HRs for overall and cardiovascular mortality were observed among vitamin D deficient obese/abdominally obese subjects, while for cancer mortality among vitamin D deficient normal weight/non-abdominally obese subjects. In stratified analyses, regarding all-cause mortality, there was an additive effect of the vitamin D both insufficiency and deficiency in all BMI/WC categories. Regarding cardiovascular mortality, there was an additive effect of vitamin D deficiency in all BMI/WC categories, but the additive effect of vitamin D insufficiency reached significance only in normal weight subjects. Regarding cancer mortality, the effect did not reach significance among obese subjects for vitamin D deficiency, while for insufficiency, significance was reached only among non-abdominally obese subjects. Interestingly, vitamin D surplus was associated with increased risk for cancer mortality in obese subjects, but there was an inadequate number of subjects in this category to make proper judgment. In conclusion, vitamin D insufficiency and deficiency gradually increase risk for mortality across all BMI/WC categories. In our analyses, vitamin D deficiency overcame the effect of obesity on mortality rates.

## Introduction

Vitamin D, as an essential micronutrient, has pleiotropic skeletal and non-skeletal actions, including its anti-inflammatory, anti-proliferative, anti-oxidative, and immunomodulatory effects (1). An increasing number of studies have shown that vitamin D deficiency is associated with obesity and metabolic disorders related to obesity (2–4). Compared with normal weight subjects, obese subjects are more likely to have vitamin D deficiency, most probably due to volumetric dilution, even though other mechanisms also could have a role (5, 6). Currently, hypovitaminosis D is observed at high rates, most probably because of the modern lifestyle, but some argue that this may be also due to the high global obesity prevalence (5, 7).

Obesity and vitamin D deficiency are now considered major public health problems worldwide (2). Recent meta-analyses and systematic review studies proposed that vitamin D deficiency may be associated with mortality, including all-cause mortality and cause-specific mortality (8–10). According to previous studies, vitamin D deficiency may be more significantly associated with cardiovascular diseases (CVD) and was also closely related to some carcinoma types (11–13). Low levels of circulating 25-hydroxy-vitamin D [25(OH)D] may be associated with an increased risk of CVD, especially recurrent CVD events and CVD mortality (14). Meanwhile, the global prevalence of obesity is also rising, and CVD is one of the main causes of death among the obese (15). Additionally, obesity or serum vitamin D level may affect the severity or mortality of some diseases. For example, some research results showed that the incidence and mortality of novel coronavirus disease 2019 (COVID-19) were higher in several European countries with high latitude or high obesity prevalence (16). In some specific populations, such as menopausal women, it has been shown that the relationship between vitamin D levels and mortality may differ among participants with different body mass index (BMI) or waist circumference (WC), but there was no similar conclusion in the general population (17, 18). These studies all implied that vitamin D or obesity not only independently have an effect on mortality, but also there may be a potential additive effect between them. However, the potential additive effect of serum vitamin D status and obesity on mortality has not been well-studied.

We aimed to examine in detail the possible additive effect of obesity and vitamin D status [assessed through serum 25(OH)D concentration] with regard to all-cause and cause-specific mortality, using the data from National Health and Nutrition Examination Surveys (NHANES), large scale surveys conducted in the United States (19).

## Materials and methods

The National Health and Nutrition Examination Survey (NHANES) is a nationally representative survey administered by the National Center for Health Statistics division of the Centers for Disease Control and Prevention and US Department of Agriculture. The NHANES utilizes a multistage, stratified area probability sampling design to select participants representative of the US population, and combines in-person interviews and physical examinations *via* a mobile examination center to collect data (20). NHANES was approved by the institutional review board of the National Center of Health Statistics. All participants provided written informed consent.

Data were analyzed from NHANES III (1988–1994) and NHANES 2001–2014. The average follow-up time was 11.9 years. Considering age-related reductions in lean muscle mass and subclinical disorders which may affect body weight, the analysis samples were limited to participants aged 20–79 years, without pregnancy and with complete data on BMI, WC, serum 25(OH)D concentrations and survival during follow-up (21).

### Body measurements

In the current study, we classified participants as underweight (BMI < 18.5 kg/m^2^), normal weight (BMI = 18.5–24.9 kg/m^2^), overweight (BMI = 25.0–29.9 kg/m^2^), and obese (BMI ≥ 30 kg/m^2^) groups according to BMI (22). Abdominal obesity was defined as WC ≥ 102 cm for men and WC ≥ 88 cm for women (23).

### Serum 25(OH)D concentration

Serum 25(OH)D concentrations are considered to be the most reliable index of vitamin D status. Serum 25(OH)D concentrations were measured by DiaSorin radioimmunoassay kit (Stillwater, MN) in the NHANES III (1988–1994) and NHANES 2001–2006, and then were determined by a standardized liquid chromatography–tandem mass spectrometry (LC-MS/MS) method (24). Serum [25(OH)D] data from NHANES III (1988–1994) and NHANES 2001–2006 have been converted by using regression to equivalent 25(OH)D measurements from a standardized LC-MS/MS method and we used the LC-MS/MS-equivalent data for all analyses as recommended by analytical guidelines (24). According to serum25(OH)D concentrations and the US Institute of Medicine (IOM) guidelines from 2011, the vitamin D status was categorized into deficiency (<12.0 ng/mL), insufficiency (12.0–19.9 ng/mL), sufficiency (20.0–50 ng/mL) and possibly harmful (>50 ng/ml) (25). Nevertheless, according to the Endocrine Society guidelines form 2011, vitamin D deficiency was defined as serum levels of 25(OH)D < 20 ng/mL, insufficiency as levels 20–30 ng/mL, while levels ≥ 30 ng/ml were characterized as the normal range (26). In our study we used the more strict IOM definitions (27).

### Outcomes

Mortality related information for all causes and specific diseases was obtained by linking to the National Death Index as at December 31, 2015. Outcomes were classified using ICD-10 codes. CVD mortality codes include: ICD-10 codes I00–I09, I11, I13, I20–I51, or I60–I69, and cancer mortality codes include: ICD-10 codes C00–C97. Because the deaths due to CVD were not available on US National Death Index matched mortality dataset after December 31, 2011, we only included participants from NHANES III and NHANES 2001–2010 for CVD mortality.

### Covariates

In the surveys, the self-reported data were collected on age, race/ethnicity, gender, educational level, ratio of family income to poverty, leisure-time physical activity, smoking, and drinking (3). Leisure-time physical activity was divided into three groups: inactive group (no leisure time physical activity), moderately active group (leisure time moderate physical activity 1–5 times per week or leisure-time vigorous physical activity 1–3 times per week), active group: more moderate or vigorous leisure time physical activity than above (28). The smoking status was categorized into “never,” “former,” or “current smoker.” Never smoker was defined as smoking never or less than 100 cigarettes in life. Former smoker was defined as smoking more than 100 cigarettes in life but no smoking temporally, while current smokers were those who reported temporally smoking cigarettes “every day” or “some days” (29). Diet and alcohol consumption related data were from 24-h dietary recalls (only day 1 recall was included). Drinking was grouped according to the dietary guidelines for American residents (30).

### Statistical analysis

For continuous and categorical variables, respectively, the analysis of variance and Pearson χ2 test were performed. In addition, the Bonferroni method *post-hoc* tests were made in analysis of variance, and *P*-value was corrected for multiple comparisons by the Bonferroni method. A *post-hoc* power analysis was also performed in our study and the result was satisfactory (power > 0.9). We used multiple imputation based on chained equations (MICE) to impute the missing data of covariates, but we also performed analyses with excluded all subjects who miss any of the main covariates (data presented in Supplementary material).

In our study, Cox proportional hazard regression models were used to estimate the hazard ratios (HR) and 95% confidence intervals (95% CI) for outcomes. Due to the rare number of underweight participants, we only performed descriptive statistics for them. The models were adjusted age, race/ethnicity, gender, educational level, family income to poverty ratio, leisure-time physical activity, smoking and drinking. To see the additive effects on mortality, we stratified Cox Hazard Ratios (HRs) across different categories of BMI/WC and vitamin D status. Reference groups were vitamin D sufficient, normal weight and not abdominally obese. Additionally, in supplementary multivariate models in Supplementary Table 4 (Supplementary Models 1–3) we also included as covariates dietary supplement use, polyunsaturated fatty acid intake, calcium and magnesium intake (Supplementary Table 4 in Supplementary Model 1), healthy eating index (HEI) (Supplementary Table 4 in Supplementary Model 2) or specific foods intake in HEI scores (Supplementary Table 4 in Supplementary Model 3) (HEI-1995 for NHANES 1988–1994 and HEI-2015 for NHANES 2001–2014), since those dietary factors have been shown to interact with associations of vitamin D status with mortality (31–34). Considering that vitamin D status is associated with renal function, we also further adjusted our main model for kidney function, estimated by the glomerular filtration rate (Supplementary Table 4 in Supplementary Model 4) (35). We also repeated the main analyses after excluding the participants with missing data on any of the main covariates (age, gender, race, educational level, ratio of family income to poverty, leisure-time physical activity, smoking, and drinking). All analyses were performed using Stata software (version 16). Gpower software (version 3.1) was used for power analysis. Two-sided *P* < 0.05 was considered for statistical significance.

## Results

### Population characteristics

The baseline characteristics of the participants form NHANES III and NHANES 2001–2014 were shown in Table 1. The average follow-up time was 11.9 years. We included 40058 participants aged 20–79 who were not pregnant and had no missing data on BMI, WC, serum 25(OH)D concentrations and survival. The flow chart and the comparison of baseline characteristics between excluded and included participants can be seen in Supplementary Figure 1 and Supplementary Table 1, respectively.

**TABLE 1:** Baseline characteristics of participants from US National Health and Nutrition Examination Survey (US NHANES) III and 2001–2014.

Characteristics	Total	Underweight	Normal weight	Overweight	Obese
	*N* = 40058	*N* = 648 (1.6)	*N* = 12309 (30.7)	*N* = 13731 (34.3)	*N* = 13370 (33.4)
Mean age in years*	46.9 (46.8–47.1)	41.2 (39.8–42.6)	43.5 (43.2–43.8)	48.6 (48.3–48.9)	48.7 (48.4–48.9)
Gender*					
Male	19586 (48.9/100)	226 (34.9/1.2)	5794 (47.1/29.6)	7810 (56.9/39.9)	5756 (43.1/29.4)
Female	20472 (51.1/100)	422 (65.1/2.1)	6515 (52.9/31.8)	5921 (43.1/28.9)	7614 (57.0/37.2)
Race/ethnicity*					
Non-Hispanic white	17770 (44.4/100)	325 (74.0/1.8)	5883 (75.8/33.1)	6035 (73.5/34.0)	5527 (69.0/31.1)
Non-Hispanic black	9255 (23.1/100)	170 (11.4/1.8)	2514 (8.4/27.2)	2855 (9.6/30.9)	3716 (14.6/40.2)
Mexican American	8526 (21.3/100)	68 (2.9/0.8)	2240 (5.1/26.3)	3338 (7.8/39.4)	2880 (8.3/33.8)
Other	4507 (11.3/100)	85 (11.7/1.9)	1672 (10.7/37.1)	1503 (9.1/33.4)	1247 (8.2/27.7)
Education*					
Less than high school	13610 (34.0/100)	216 (33.3/1.6)	3724 (30.3/27.4)	4966 (36.2/36.5)	4704 (35.2/34.6)
High school or equivalent	12068 (30.1/100)	200 (30.9/1.7)	3758 (30.6/31.1)	4045 (29.5/33.5)	4065 (30.4/33.7)
College or above	14365 (35.9/100)	232 (35.8/1.6)	4821 (39.2/33.6)	4717 (34.4/32.8)	4595 (34.4/32.0)
Family income-poverty ratio*					
≤1.0	8916 (23.3/100)	192 (31.4/2.2)	2717 (22.9/30.5)	2914 (22.2/32.7)	3093 (24.3/34.7)
1.0–3.0	15707 (41.0/100)	259 (42.4/1.7)	4678 (39.4/29.8)	5330 (40.6/33.9)	5440 (42.6/34.6)
>3.0	13721 (35.8/100)	160 (26.2/1.2)	4468 (37.7/32.6)	4868 (37.1/35.5)	4225 (33.1/30.8)
Leisure-time physical activity*					
Inactive	16972 (47.0/100)	304 (50.1/1.8)	4790 (42.4/28.2)	5576 (45.1/32.9)	6302 (53.1/37.1)
Moderately active	12184 (33.8/100)	163 (28.0/1.3)	3902 (34.6/32.0)	4271 (34.5/35.1)	3848 (32.4/31.6)
Active	6945 (19.2/100)	116 (19.9/1.7)	2595 (23.0/37.4)	2518 (20.4/36.3)	1716 (14.5/24.7)
Smoking*					
Never	20630 (51.5/100)	287 (46.5/1.4)	6250 (49.0/30.3)	6890 (48.9/33.4)	7203 (50.9/34.9)
Former	9758 (24.4/100)	68 (10.3/0.7)	2402 (20.8/24.6)	3731 (27.9/38.2)	3557 (28.0/36.5)
Current	9657 (24.1/100)	293 (43.2/3.0)	3654 (30.2/37.8)	3107 (23.2/32.2)	2603 (21.2/27.0)
Alcohol, g/d*					
<14	32159 (81.3/100)	495 (76.6/1.5)	9473 (75.9/29.5)	10840 (76.7/33.7)	11351 (84.4/35.3)
14–28	2745 (6.9/100)	61 (10.8/2.2)	962 (8.3/35.1)	999 (8.2/36.4)	723 (5.9/26.3)
≥ 28	4670 (11.8/100)	83 (12.6/1.8)	1717 (15.8/36.8)	1734 (15.1/37.1)	1136 (9.7/24.3)
BMI, kg/m^2^*	28.4 (28.3–28.5)	17.5 (17.4–17.6)	22.4 (22.4–22.5)	27.4 (27.3–27.4)	35.5 (35.4–35.6)
Waist circumference, cm*	96.9 (96.7–97.1)	70.5 (70.1–70.9)	82.4 (82.2–82.5)	95.8 (95.7–96.0)	112.7 (112.4–112.9)
Abdominal obesity*					
Not abdominally obese	19511 (48.7/100)	646 (99.7/3.3)	11354 (92.2/58.2)	6939 (50.5/35.6)	572 (4.3/2.9)
Abdominally obese	20547 (51.3/100)	2 (0.3/0.0)	955 (7.8/4.7)	6792 (49.5/33.1)	12798 (95.7/62.3)
Vitamin D status, ng/mL*	23.6 (23.5–23.7)	24.3 (23.4–25.2)	25.3 (25.1–25.4)	24.0 (23.9–24.2)	21.7 (21.5–21.8)
Vitamin D status*					
Sufficiency	24111 (60.2/100)	381 (67.4/1.6)^a, b^	8074 (75.5/33.5)^a, c^	8651 (71.8/35.9)^b, d^	7005 (60.4/29.1)^c, d^
Insufficiency	11662 (29.1/100)	161 (20.3/1.4) ^a^	3040 (17.8/26.1)^b, c^	3895 (22.1/33.4)^b, d^	4566 (30.1/39.2)^a, c, d^
Deficiency	3807 (9.5/100)	90 (9.6/2.4)^a, b^	976 (4.7/25.6)^a, c, d^	1041 (4.7/27.3)^c, e^	1700 (8.7/44.7)^b, d, e^
Possibly harmful	478 (1.2/100)	16 (2.7/3.4)^a, b^	219 (2.0/45.8)^c, d^	144 (1.3/30.1)^a, c, e^	99 (0.8/20.7)^b, d, e^
**Death**					
All-cause mortality*	6617 (16.5/100)	133 (20.5/2.0)^a, b^	1949 (15.8/29.5)^a, c^	2395 (17.4/36.2)^c, d^	2140 (16.0/32.3)^b, d^
CVD mortality	1320 (3.3/100)	17 (2.6/1.3)	365 (3.0/27.7)	476 (3.5/36.1)	462 (3.5/35.0)
Cancer mortality	1614 (4.0/100)	34 (5.3/2.1)	491 (4.0/30.4)	589 (4.3/36.5)	500 (3.7/31.0)

Data are N (% by column/% by row) or mean (95% CI). *There were statistical differences among different BMI categories (*P* < 0.001). *P*-values were calculated using analysis of variance with *post-hoc* Bonferroni test and χ2 test for continuous and categorical variables, respectively. The same superscript indicates statistically significant difference between the two groups (*P* < 0.05). *P*-values have been corrected by the Bonferroni method. CVD, cardiovascular disease; BMI, body mass index.

Among the 40,058 participants, just 1.6% were underweight, while other BMI categories were quite equally represented. However, there were significant gender differences regarding obesity and overweight prevalence: among females, the majority were obese (37.2%), and among males, the majority were overweight (39.9%). The highest proportion of obese was among non-Hispanic Blacks (40.2%), then Mexican American (33.8%), while the highest proportion of overweight was among Mexican American (39.2%), then non-Hispanic Whites (34.0%). In total, both Mexican Americans and non-Hispanic Blacks had the highest proportion of subjects with overweight/obesity. Interestingly, regarding education and family income-poverty ratio, there was no huge difference, but regarding leisure time physical activity, smoking and alcohol consumption, the lowest proportion of obese was among physically active, current smokers and those who consumed more that 14 g alcohol per day.

Abdominal obesity was present in half of the overweight subjects and in almost all of the obese subjects (95.7%).

In total, vitamin D deficiency [serum 25(OH)D < 12 ng/mL] was present in 6.0% of subjects, while insufficiency [serum 25(OH)D 12.0–19.9 ng/mL] was present in 22.9% of subjects. Among vitamin D deficient and insufficient subjects, there was the highest proportion of obese subjects (44.7 and 39.2%, respectively), then overweight subjects (27.3 and 33.4%, respectively), which means that among vitamin D deficient and insufficient subjects, respectively, 72.0 and 72.6% were with BMI ≥ 25 kg/m^2^. Interestingly, in both obese and underweight subjects, the proportion of vitamin D deficiency or insufficiency was higher than in normal weight or overweight group.

### Multivariate analysis of association between vitamin D status and all-cause and cause-specific mortality among different BMI/WC groups

First, we analyzed the association of obesity levels or vitamin D status with mortality in the total sample (Tables 2, 3). The results indicated that obesity, abdominal obesity, vitamin D insufficiency and vitamin D deficiency were associated with increased risk for all-cause mortality and CVD mortality. Interestingly and unexpectedly, all-cause and CVD mortality HRs for vitamin D deficiency/insufficiency were higher than HRs for obesity/abdominal obesity. Vitamin D deficiency was associated with the highest risk for all-cause and CVD mortality. Interestingly, overweight had a protective effect on all-cause mortality. Regarding cancer-mortality, only vitamin D insufficiency and deficiency were associated with the increased risk. We also provide the HRs of all included covariates for all-cause and cause-specific mortality in the models which included both vitamin D status and BMI or WC categories (Supplementary Tables 2, 3). Results were very similar, with again much higher HRs for vitamin D status vs. obesity status regarding all-cause and CVD mortality, with vitamin D deficiency bringing the highest risk. For cancer-mortality, only vitamin D status was a significant predictor, with both insufficiency and deficiency contributing to the higher risk (but deficiency contributed more). Adjusting for obesity level negligibly changed significance for vitamin D status HRs. Regarding other covariates, only leisure-time physical activity was not associated with mortality rates. Smoking brought the highest risk for all three mortalities (Supplementary Tables 2, 3).

**TABLE 2:** The associations of different obesity levels with all-cause and cause-specific mortality in NHANES III and NHANES 2001–2014: HRs (95% CIs) for different BMI categories.

Death	HR (95% CI)	*P*-values
**All-cause mortality**		
Normal weight	Reference	
Overweight	0.91 (0.86–0.97)*	0.003
Obesity	1.08 (1.01–1.15)*	0.017
Non-abdominal obesity	Reference	
Abdominal obesity	1.09 (1.03–1.15)*	0.001
**CVD mortality**		
Normal weight	Reference	
Overweight	0.93 (0.81–1.07)	0.291
Obesity	1.25 (1.09–1.44)*	0.002
Non-abdominal obesity	Reference	
Abdominal obesity	1.28 (1.14–1.44)*	<0.001
**Cancer mortality**		
Normal weight	Reference	
Overweight	0.92 (0.81–1.04)	0.162
Obesity	1.02 (0.90–1.16)	0.779
Non-abdominal obesity	Reference	
Abdominal obesity	0.97 (0.88–1.08)	0.623

All models were adjusted for age, gender, race/ethnicity, educational level, family income to poverty ratio, leisure-time physical activity, smoking, and drinking. *P < 0.05. HR, hazard ratio; CI, confidence interval; BMI, body mass index; CVD, cardiovascular disease.

**TABLE 3:** The associations of serum vitamin D status with all-cause and cause-specific mortality in NHANES III and NHANES 2001–2014: HRs (95% CIs) for different vitamin D status categories.

Death	HR (95% CI)	*P*-values
**All-cause mortality**		
Sufficiency	Reference	
Insufficiency	1.18 (1.12–1.25)*	<0.001
Deficiency	1.48 (1.36–1.62)*	<0.001
Possibly harmful	1.00 (0.65–1.54)	1.000
**CVD mortality**		
Sufficiency	Reference	
Insufficiency	1.25 (1.10–1.42)*	0.001
Deficiency	1.65 (1.35–2.00)*	<0.001
Possibly harmful	–	–
**Cancer mortality**		
Sufficiency	Reference	
Insufficiency	1.18 (1.05–1.32)*	0.005
Deficiency	1.42 (1.19–1.69)*	<0.001
Possibly harmful	1.56 (0.80–3.01)	0.189

All models were adjusted for age, gender, race/ethnicity, educational level, family income to poverty ratio, leisure-time physical activity, smoking, and drinking. *P < 0.05. HR, hazard ratio; CI, confidence interval; CVD, cardiovascular disease.

Next, we performed analyses to see the additive effects of vitamin D status and obesity levels, by comparing with the risk in “normal weight, vitamin D sufficient” subjects. In the whole sample analyses, there was the highest risk of all-cause and CVD mortality in participants with both obesity and vitamin D deficiency (Table 4). It is observable that through all BMI/WC categories, with deterioration of the vitamin D status, the risk for all-cause and CVD mortality gradually increases. The highest impact of joint vitamin D deficiency and obesity/abdominal obesity was on CVD mortality. Interestingly, the impact of vitamin D deficiency much overcame the effect of obesity for all-cause and CVD mortality. Actually, for all-cause mortality, among vitamin D sufficient subjects the effect of obesity was not significant, while overweight even had a protective effect. Regarding cancer mortality, only vitamin D status was associated with the increased risk, and on the highest risk were vitamin D deficient normal weight/not-abdominally obese subjects, then vitamin D deficient overweight subjects.

**TABLE 4:** The interaction effect of serum vitamin D status and obesity levels on all-cause and cause-specific mortality in NHANES III and NHANES 2001–2014: HRs (95% CIs) across different vitamin D status and obesity sub-categories.

	Vitamin D sufficiency	Vitamin D insufficiency	Vitamin D deficiency	Possibly harmful
	HR (95% CI)	HR (95% CI)	HR (95% CI)	HR (95% CI)
**All-cause mortality**				
**BMI**				
Normal weight	Reference	1.19 (1.08–1.32)*	1.51 (1.30–1.76)*	0.80 (0.38–1.68)
Overweight	0.91 (0.84–0.99)*	1.08 (0.99–1.19)	1.33 (1.14–1.54)*	1.07 (0.51–2.25)
Obesity	1.08 (0.99–1.17)	1.24 (1.13–1.36)*	1.53 (1.33–1.75)*	1.25 (0.56–2.79)
**WC**				
Non-Abdominal obesity	Reference	1.22 (1.12–1.33)*	1.55 (1.36–1.77)*	0.95 (0.49–1.82)
Abdominal obesity	1.10 (1.03–1.18)*	1.26 (1.17–1.36)*	1.57 (1.41–1.76)*	1.16 (0.66–2.05)
**CVD mortality**				
** BMI**				
Normal weight	Reference	1.38 (1.10–1.74)*	1.59 (1.11–2.28)*	–
Overweight	0.98 (0.82–1.17)	1.08 (0.87–1.35)	1.70 (1.22–2.37)*	–
Obesity	1.25 (1.04–1.52)*	1.56 (1.27–1.92)*	2.07 (1.53–2.80)*	–
**WC**				
Non-Abdominal obesity	Reference	1.30 (1.07–1.58)*	1.78 (1.31–2.42)*	–
Abdominal obesity	1.31 (1.13–1.53)*	1.56 (1.31–1.85)*	2.00 (1.56–2.58)*	–
**Cancer mortality**				
** BMI**				
Normal weight	Reference	1.26 (1.03–1.54)*	1.60 (1.19–2.14)*	1.22 (0.39–3.83)
Overweight	0.94 (0.80–1.10)	1.09 (0.90–1.31)	1.49 (1.12–1.99)*	1.15 (0.29–4.62)
Obesity	1.06 (0.89–1.26)	1.22 (1.01–1.48)*	1.24 (0.93–1.66)	3.11 (1.16–8.37)*
**WC**				
Non-Abdominal obesity	Reference	1.25 (1.06–1.47)*	1.50 (1.16–1.93)*	1.07 (0.34–3.35)
Abdominal obesity	1.00 (0.87–1.14)	1.13 (0.96–1.31)	1.37 (1.09–1.72)*	1.94 (0.77–4.89)

All models adjusted for age, gender, race/ethnicity, educational level, family income to poverty ratio, leisure-time physical activity, smoking, and drinking. *P < 0.05. HR, hazard ratio; CI, confidence interval; BMI, body mass index; WC, waist circumference; CVD, cardiovascular disease.

**FIGURE 1:**
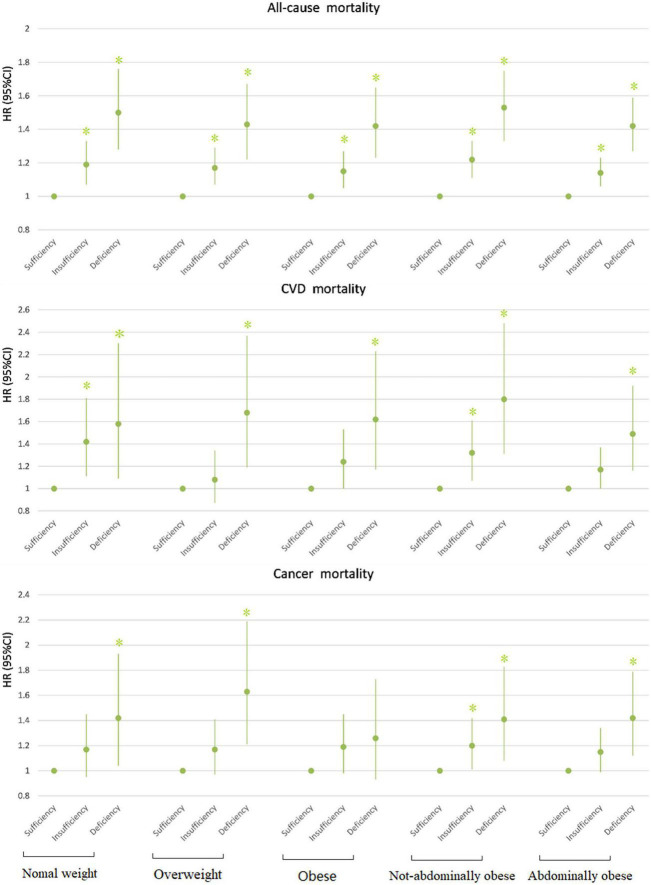
Association between 25(OH)D concentration and mortality by BMI/WC. All models adjusted for age, race, gender, educational level, ratio of family income to poverty, leisure-time physical activity, smoking, and drinking. *P < 0.05. HR, hazard ratio; CI, confidence interval; CVD, cardiovascular disease.

We further stratified the association between vitamin D status and mortality by different BMI/WC categories, with adjustments for age, race, gender, educational level, family income to poverty ratio, leisure-time physical activity, smoking, and drinking, to see in which BMI/WC category the effect of vitamin D deficiency/insufficiency will be the highest (Tables 5, 6 and Figure 1).

**TABLE 5:** Stratified HRs (95% CIs) across different BMI categories and additive effect of serum 25(OH)D status on all-cause and cause-specific mortality in NHANES III and NHANES 2001–2014.

Vitamin D status	BMI Hazard ratio (95% CIs)								
	Normal weight			Overweight			Obese		
**All-cause mortality**	**n**		***P***	**n**		***P***	**n**		***P***
Number of deaths		1,949			2,395			2,140	
Sufficiency	1,160	1 (Reference)		1,403	1 (Reference)		1,037	1 (Reference)	
Insufficiency	577	1.19 (1.07–1.33)*	0.001	777	1.17 (1.07–1.29)*	0.001	817	1.15 (1.05–1.27)*	0.004
Deficiency	205	1.50 (1.28–1.76)*	<0.001	208	1.43 (1.22–1.67)*	<0.001	280	1.42 (1.23–1.65)*	<0.001
Possibly harmful	7	1.15 (0.38–3.54)	0.800	7	1.16 (0.55–2.45)	0.693	6	1.26 (0.56–2.82)	0.573
** CVD mortality**									
Number of deaths		362			468			450	
Sufficiency	210	1 (Reference)		286	1 (Reference)		219	1 (Reference)	
Insufficiency	116	1.42 (1.11–1.81)*	0.005	138	1.08 (0.87–1.34)	0.500	173	1.24 (1.00–1.53)	0.050
Deficiency	36	1.58 (1.09–2.30)*	0.017	44	1.68 (1.19–2.37)*	0.003	58	1.62 (1.17–2.23)*	0.003
Possibly harmful	0	–		0	–		0	–	
** Cancer mortality**									
Number of deaths		491			589			500	
Sufficiency	281	1 (Reference)		340	1 (Reference)		243	1 (Reference)	
Insufficiency	151	1.17 (0.95–1.45)	0.150	188	1.17 (0.97–1.41)	0.100	195	1.19 (0.98–1.45)	0.086
Deficiency	56	1.42 (1.04–1.93)*	0.027	59	1.63 (1.21–2.19)*	0.001	58	1.26 (0.93–1.73)	0.141
Possibly harmful	3	1.16 (0.37–3.64)	0.800	2	1.22 (0.30–4.90)	0.784	4	3.18 (1.18–8.61)*	0.023

All models were adjusted for age, race, gender, educational level, ratio of family income to poverty, leisure-time physical activity, smoking, and drinking. *P < 0.05. HR, hazard ratio; CI, confidence interval; BMI, body mass index; CVD, cardiovascular disease.

**TABLE 6:** Stratified HRs (95% CIs) across different WC categories and additive effect of serum 25(OH)D status on all-cause and cause-specific mortality in NHANES III and NHANES 2001–2014.

Vitamin D status	WC Hazard ratio (95% CIs)					
	Not abdominally obese			Abdominally obese		
**All-cause mortality**	**n**		***P***	**n**		***P***
Number of deaths		2,848			3,769	
Sufficiency	1,679	1 (Reference)		1,985	1 (Reference)	
Insufficiency	885	1.22 (1.11–1.33)*	<0.001	1,330	1.14 (1.06–1.23)*	0.001
Deficiency	275	1.53 (1.33–1.75)*	<0.001	442	1.42 (1.27–1.59)*	0.001
Possibly harmful	9	0.86 (0.45–1.66)	0.660	12	1.13 (0.64–1.99)	0.683
** CVD mortality**						
Number of deaths		509			788	
Sufficiency	298	1 (Reference)		426	1 (Reference)	
Insufficiency	160	1.32 (1.07–1.61)*	0.009	275	1.17 (1.00–1.37)	0.058
Deficiency	51	1.80 (1.31–2.48)*	<0.001	87	1.49 (1.16–1.92)*	0.002
Possibly harmful	0	–		0	–	
** Cancer mortality**						
Number of deaths		746			868	
Sufficiency	430	1 (Reference)		454	1 (Reference)	
Insufficiency	239	1.20 (1.01–1.42)*	0.033	304	1.15 (0.99–1.34)	0.075
Deficiency	74	1.41 (1.08–1.83)*	0.011	104	1.42 (1.12–1.79)*	0.003
Possibly harmful	3	1.01 (0.32–3.17)	0.980	6	2.10 (0.93–4.73)	0.072

All models were adjusted for age, race, gender, educational level, ratio of family income to poverty, leisure-time physical activity, smoking, and drinking. *P < 0.05. HR, hazard ratio; CI, confidence interval; CVD, cardiovascular disease; WC, waist circumference.

In all BMI- and WC-categories, both insufficiency and deficiency of vitamin D were additional risk factors for all-cause mortality. The participants with vitamin D deficiency had the most increased risk of all-cause mortality compared with those with vitamin D sufficiency, across all categories of BMI and WC. The participants with vitamin D insufficiency had also the significantly higher risk of all-cause mortality compared with vitamin D sufficiency, but the risk was lower compared with vitamin D deficiency (Tables 5, 6). Across all BMI/WC categories, the risk vitamin D insufficiency/deficiency quite equally increased, but seems that highest effect was among normal weight and not abdominally obese subjects (Figure 1).

For CVD mortality, our data showed that the insufficient vitamin D status was an additional risk factor only in the normal weight and non-abdominally obese subjects, while in obese and abdominally obese subjects it only approached statistical significance. In contrast, vitamin D deficiency was an additional risk factor for CVD mortality in all BMI and WC-subgroups (Tables 5, 6 and Figure 1).

Regarding cancer mortality, the additive effect of vitamin D deficiency was seen in all BMI/WC categories, except in obese subjects, while the additive effect of vitamin D insufficiency was seen only in non-abdominally obese subjects. Interestingly, there was an additive effect of vitamin D surplus (levels above 50 ng/ml) and obesity on the risk for cancer mortality (Table 4). Actually, only in obese subjects the vitamin D surplus was associated with the increased risk for cancer mortality (Table 5). This observation is interesting and requires further exploration, but due to particularly small number of subjects in that category (only 4), it is not possible to draw proper concussion.

The results of our supplementary models were consistent with the results of the presented main models (Supplementary Table 4), with difference that the effect of vitamin D insufficiency on was also observed in obese and abdominally obese subjects. We also repeated analyses in the main models after excluding the participants with missing any of the main covariates, and the results were very similar, with only difference that the effect of vitamin D deficiency was no more seen in obese and abdominally-obese subjects for CVD mortality, nor was seen the effect of vitamin D surplus on cancer mortality (Supplementary Tables 5–8).

## Discussion

### Overall mortality

Our study, based on a large prospective cohort, indicated that lower levels of serum vitamin D were significantly associated with higher all-cause mortality. This was consistent with some data from previous research (36, 37). Additionally, there was an additive effect of the vitamin D both insufficiency and deficiency on all-cause mortality in all BMI/WC categories. It is well-known that obesity is one of the main risk factors for pre-mature death, and among examined 87 risk factors for pre-mature death, high BMI was on the fifth place, according to the Global burden of disease study report from 1990–2019 (38). Therefore, we expected that obesity will have much more significant impact on overall mortality, compared with vitamin D status. Nevertheless, the results from this study have shown quite the opposite: the impact of vitamin D status overcome the impact of overweight and obesity, since in our Cox regression models for overall mortality, HRs for vitamin D both insufficiency and deficiency were much higher, compared with HRs for overweight and obesity, which was an expected finding (actually, overweight had a protective effect). The explanations for the additive effects of obesity and vitamin D deficiency on overall mortality are not clear. Since CVD mortality was even more connected with additive effect of vitamin D status and obesity, it could be projected the effect on overall mortality was mostly conveyed through the effect on CVD mortality. In our study, CVD mortality represented only about one fifth of total mortality, but we did not include all surveys for assessing CVD mortality, and proportion of this mortality must be higher. Additionally, mortality of many other diseases and conditions can be associated with vitamin D status and obesity, e.g., mortality from respiratory diseases, infective diseases or some other chronic diseases, including endocrine (diabetes), neurological, kidney diseases, rheumatoid diseases, etc. (37). The mechanisms of the association between vitamin D and obesity can be multiple. Not only is there a simple dilution of vitamin D by increased fat deposits and plasma volume, but also some studies have indicated that in obesity the rate of uptake up vitamin D in adipose tissue may be increased, together with the increased rate of its inactivation/catabolism and elimination, and decreased rate of synthesis and absorption (6, 39, 40). Therefore, obese participants may need more vitamin D in order to maintain adequate status of serum vitamin D (5, 41, 42). With higher sequestration of vitamin D in adipose tissue, there will be much lower bioavailability of vitamin D for many other tissues, and it is known that vitamin D has numerous physiological effects (apart from the effect on calcium and phosphorus metabolism in bones and risk for fractures), including hormonal, metabolic, anti-oxidative, anti-proliferative, anti-infective, and immuno-modulatory actions. All these mechanisms may explain the higher risk for overall mortality in participants with both obesity and vitamin D deficiency.

### Cardiovascular diseases mortality

With respect to CVD mortality, our results showed again that there was a strong additive effect of vitamin D deficiency and obesity, since participants with both obesity and vitamin D deficiency had the highest risk for CVD mortality, compared with other categories: almost 2.1 times higher than in vitamin D sufficient normal weight subjects. In contrast, obesity alone, or vitamin D deficiency alone, had only 1.3 and 1.6 times increased risk, respectively. Interestingly and unexpectedly, the effect of overweight alone on CVD mortality was not observed. In stratified analyses, in all BMI/WC categories there was the additive effect of vitamin D deficiency, but for vitamin D insufficiency, the significant additive effect was only seen in normal weight and non-abdominally obese subjects. The possible reasons are that the effect of overweight/obesity and abdominal obesity overcomes the effect of vitamin D insufficiency on CVD mortality. Moreover, for CVD mortality we only included participants from NHANES III and NHANES 2001–2010, so the smaller number of participants probably did not allow to reach significance, even though the additive effect was still present. Additionally, there can be a cut-off level in vitamin D levels, under which an additive effect of vitamin D insufficiency on the risk for CVD mortality can be observed (37). Similarly, as for the all-cause mortality, with higher sequestration of vitamin D in adipose tissue, there will be much lower bioavailability of vitamin D for other tissues, which may have adverse effects on cardiovascular system, since vitamin D was shown as potent regulator of its components, despite some controversy on the particular mechanisms and outcomes (43–46). Our results on the effect of vitamin D status on CVD mortality are in accordance with the data from the recent systematic review by Heat et al. (47), which showed the evidence for the association in observational studies. However, the data from intervention studies did not show a significant effect, which indicates a probable confounding effect in observational studies (47).

### Cancer mortality

It is worth noting that in our study, we observed the effect of vitamin D both deficiency and insufficiency on increased risk for cancer mortality, but the effect of obesity was not observed. In the whole sample, the highest increased risk was seen among vitamin D deficient normal weight/not-abdominally obese participants, then overweight participants. In the stratified analyses, the effect of vitamin D insufficiency/deficiency did not reach statistical significance in some weight categories, probably due to small numbers included. Interestingly, there was an additive effect of vitamin D surplus on the increased risk for cancer mortality among obese participants, both in whole sample and stratified analyses. However, because of small number of these participants (only 4), there could not be reliable conclusions and additional studies are needed. Our data are in accordance with the results from other studies, which show that vitamin D both deficiency and surplus may be associated with the increased risk for cancer mortality (37, 48–52). Better vitamin D status was associated with reduced mortality for breast cancer, colorectal cancer, prostate cancer, pancreatic cancer and hematological malignancies (37). Trials with vitamin D3 supplementation also show potential effect on reduced cancer mortality (53, 54). Interestingly, data show that vitamin D status was not associated with the increased risk for cancer incidence, indicating that vitamin D status might be more involved in cancer progression than initial cancerogenesis (37).

### Study limitation

The main limitation of this study is that BMI, WC, and vitamin D concentrations were assessed only at one time point, at the baseline of quite long follow-up, so both vitamin D and obesity status could change in the meantime and we do not have insight into their dynamics, making reliability of our associations questionable. This is a common limitation of the long-term cohort prospective studies having only one time point measurement of the examined modifiable risk factors. Second, vitamin D levels may vary with season (55), and our data did not include the season of vitamin D measurement, as very important covariate, since relevant data were not available in NHANES III (3). Moreover, we also did not include data on sunlight exposure or latitude/altitude in the analyses, but surveys indicate that despite relatively sufficient sunshine, the prevalence of vitamin D deficiency is still high in Africa, India, Australia, Asia, South America, and even the Middle East (5). Additionally, work time physical activity and physical activity when commuting to work were not included when assessing physical activity level, because the related data was only available in NHANES 2001–2014. We used only one 24 h dietary recall to obtain data on dietary and alcohol intakes, which is probably not enough to adequately assese usual intakes. Even though we included multiple covariates, probably some other covariates could be also considered (e.g., existence of certain chronic diseases at baseline, medication usage, level of stress, professional, and environmental risk factors, etc.). Although we had a quite large number of included participants, for CVD mortality we had only data from NHANES III and NHANES 2001–2010, because data on CVD mortality were not available in the US National Death Index matched mortality datasets after December 31, 2011. Additionally, we used multiple imputation to deal with the missing covariates data, which may also affect the results. To avoid this issue, we repeated our analyses with omitting subjects with missing data, and results showed just a small difference, particularly in obesity subgroups. This may be because most of the excluded participants were obese, and smaller number of the participants remaining for analyses did not allow to reach statistical significance for some analyses. Finally, the data refer to average US population aged 20–79 years, the findings may not be generalizable to other populations and beyond this age range.

## Conclusion

Our results showed that there was an additive effect of the vitamin D insufficiency/deficiency on the increased risk for all-cause and CVD mortality across all BMI/WC categories, with deficiency having much stronger effect than insufficiency. Regarding cancer mortality, vitamin D deficiency significantly increased the risk in all BMI/WC categories, except among obese subjects. The highest risk for all-cause and CVD mortality was observed among vitamin D deficient and obese/abdominally obese subjects, while for cancer mortality among vitamin D deficient and normal weight/non-abdominally obese subjects. Importantly, in our study the effect of vitamin D insufficiency overcame the effect of obesity/abdominal obesity on mortality. Maintaining sufficient serum vitamin D levels may help reduce mortality, particularly in populations at high risk. More long-term and large-scale prospective cohort studies and randomized controlled trials are required to test our findings.

## Data availability statement

The datasets presented in this study can be found in online repositories. The names of the repository/repositories and accession number(s) can be found below: https://www.cdc.gov/nchs/nhanes/index.htm. Data for this study NHANES subsample are available upon request.

## Ethics statement

The studies involving human participants were reviewed and approved by Institutional Review Board of the National Center of Health Statistics. The patients/participants provided their written informed consent to participate in this study.

## Author contributions

SS and LF: conception and design. SS, YY, XW, and DZ: data collection. SS, YY, XW, QQ, and HW: data analyses and professional drafting. SS: manuscript writing. All authors were involved in writing the manuscript and had final approval of the submitted and published versions.
