# Supplementary material for: Additive effects of obesity and vitamin D insufficiency on all-cause and cause-specific mortality
Source: Front Nutr. 2022 Oct 21;9:999489. doi: 10.3389/fnut.2022.999489 (PMC9634746; doi:10.3389/fnut.2022.999489)
Supplement: Supplementary file 1 [file Data_Sheet_1.doc]

***Supplementary Material***

**Supplementary tables and figures**

**Supplementary Table 1.** Baseline characteristics of participants included or excluded from analyses due to missing data on serum vitamin D levels, BMI, WC, and survival, and comparison between them

| Characteristics | Included  N = 40058 | excluded  N = 5522 |
| --- | --- | --- |
| Mean age in years *  Gender  Male  Female  Race/ethnicity *  Non-Hispanic white  Non-Hispanic black  Mexican American  Other  Education *  Less than high school  High school or equivalent  College or above  Family income-poverty ratio *  ≤1.0  1.0 - 3.0  >3.0  Leisure-time physical activity *  Inactive  Moderately active  Active  Smoking *  Never  Former  Current  Alcohol, g/d *  <14  14-28  ≥28  BMI, kg/m^2^ *  <18.5  18.5 – 24.9  25.0 – 29.9  ≥30  Waist circumference  Not abdominally obese  Abdominally obese | 46.9 (46.8 - 47.1)  19586 (48.9)  20472 (51.1)  17770 (44.4)  9255 (23.1)  8526 (21.3)  4507 (11.3)  13610 (34.0)  12068 (30.1)  14365 (35.9)  8916 (23.3)  15707 (41.0)  13721 (35.8)  16972 (47.0)  12184 (33.8)  6945 (19.2)  20630 (51.5)  9758 (24.4)  9657 (24.1)  32159 (81.3)  2745 (6.9)  4670 (11.8)  648 (1.6)  12309 (30.7)  13731 (34.3)  13370 (33.4)  19511 (48.7)  20547 (51.3) | 49.3 (48.9 - 49.8)  2637 (47.8)  2885 (52.3)  2232 (40.4)  1733 (31.4)  1107 (20.1)  450 (8.2)  1844 (34.0)  1888 (34.8)  1700 (31.3)  1189 (24.8)  2115 (44.2)  1483 (31.0)  2819 (55.7)  1304 (25.7)  943 (18.6)  2667 (48.4)  1342 (24.4)  1500 (27.2)  3107 (84.1)  222 (6.0)  364 (9.9)  95 (2.6)  1158 (31.9)  1121 (30.9)  1255 (34.6)  1157 (50.1)  1154 (49.9) |

Data were N (%) or mean (95%CI).

Details of exclusions due to missing analysis variables: BMI (1893), WC (3211), 25(OH)D (4110), survival (50).

Details of missing covariates among included participants: age (0), gender (0), race (0), education (15), family income-poverty ratio (1714), leisure-time physical activity (3957), smoking (13), alcohol (484)

* P<0.05 (P values were calculated using Mann-Whitney U test and χ2 test for continuous and categorical variables, respectively)

Abbreviations: BMI, body mass index; WC, waist circumference.

**Supplementary Table 2.** The HRs (95% CIs) for all covariates for all-cause and cause-specific mortality in NHANES III and NHANES 2001–2014: model including vitamin D status and BMI-categories

|  | BMI | |
| --- | --- | --- |
|  | HR (95% CI) | P values |
| **All-cause mortality**  Age  Gender  Male  Female  Race  Non-Hispanic white  Non-Hispanic black  Mexican American  Other  Education  Less than high school  High school or equivalent  College or above  Family income-poverty ratio  ≤1.0  1.0 - 3.0  >3.0  Leisure-time physical activity  Active  Moderately active  Inactive  Smoking  Never  Former  Current  Alcohol  <14 g/d  14-28 g/d  ≥28 g/d  BMI  Normal weight  Overweight  Obesity  Vitamin D status  Sufficiency  Insufficiency  Deficiency  Possibly harmful  **CVD mortality**  Age  Gender  Male  Female  Race  Non-Hispanic white  Non-Hispanic black  Mexican American  Other  Education  Less than high school  High school or equivalent  College or above  Family income-poverty ratio  ≤1.0  1.0 - 3.0  >3.0  Leisure-time physical activity  Active  Moderately active  Inactive  Smoking  Never  Former  Current  Alcohol  <14 g/d  14-28 g/d  ≥28 g/d  BMI  Normal weight  Overweight  Obesity  Vitamin D status  Sufficiency  Insufficiency  Deficiency  Possibly harmful  **Cancer mortality**  Age  Gender  Male  Female  Race  Non-Hispanic white  Non-Hispanic black  Mexican American  Other  Education  Less than high school  High school or equivalent  College or above  Family income-poverty ratio  ≤1.0  1.0 - 3.0  >3.0  Leisure-time physical activity  Active  Moderately active  Inactive  Smoking  Never  Former  Current  Alcohol  <14 g/d  14-28 g/d  ≥28 g/d  BMI  Normal weight  Overweight  Obesity  Vitamin D status  Sufficiency  Insufficiency  Deficiency  Possibly harmful | 1.08 (1.08 – 1.09) *  Reference  0.66 (0.63 – 0.70) *  Reference  0.95 (0.88 – 1.01)  0.83 (0.77 – 0.89) *  0.62 (0.55 – 0.71) *  Reference  0.84 (0.79 – 0.89)  0.78 (0.73 – 0.84) *  Reference  0.87 (0.82 – 0.93)  0.61 (0.57 – 0.66) *  Reference  0.94 (0.87 – 1.01)  1.07 (0.99 – 1.15)  Reference  1.25 (1.18 – 1.33) *  1.82 (1.70 – 1.94) *  Reference  0.91 (0.81 – 1.02)  1.08 (1.00 – 1.17) *  Reference  0.91 (0.86 – 0.97) *  1.06 (0.99 – 1.13)  Reference  1.17 (1.11 – 1.24) *  1.46 (1.34 – 1.60) *  0.98 (0.64 – 1.50)  1.10 (1.09 – 1.10) *  Reference  0.51 (0.45 – 0.58) *  Reference  0.85 (0.73 – 1.00) *  0.70 (0.59 – 0.81) *  0.56 (0.41 – 0.76) *  Reference  0.86 (0.75 – 0.99) *  0.88 (0.75 – 1.03)  Reference  0.82 (0.72 – 0.94) *  0.52 (0.44 – 0.61) *  Reference  0.90 (0.76 – 1.08)  1.15 (0.99 – 1.34)  Reference  1.31 (1.15 – 1.50) *  1.92 (1.65 – 2.23) *  Reference  0.84 (0.65 – 1.10)  0.96 (0.80 – 1.15)  Reference  0.92 (0.80 – 1.06)  1.22 (1.06 – 1.41) *  Reference  1.23 (1.08 – 1.39) *  1.61 (1.32 – 1.96) *  –  1.07 (1.07 – 1.08) *  Reference  0.71 (0.63 – 0.79) *  Reference  1.11 (0.97 – 1.26) *  0.85 (0.73 – 0.98) *  0.70 (0.54 – 0.90) *  Reference  0.92 (0.81 – 1.04)  0.87 (0.76 – 1.00)  Reference  0.86 (0.76 –0.97) *  0.71 (0.62 – 0.82) *  Reference  0.96 (0.82 – 1.11)  0.99 (0.86 – 1.13)  Reference  1.47 (1.30 – 1.67) *  2.37 (2.08 – 2.70) *  Reference  0.77 (0.61 – 0.97) *  1.03 (0.89 – 1.21)  Reference  0.91 (0.81 – 1.03)  1.00 (0.88 – 1.14)  Reference  1.18 (1.05 – 1.32) *  1.41 (1.19 – 1.68) *  1.52 (0.79 – 2.94) | <0.001  <0.001  0.099  <0.001  <0.001  <0.001  <0.001  <0.001  <0.001  0.103  0.069  <0.001  <0.001  0.090  0.038  0.002  0.083  <0.001  <0.001  0.918  <0.001  <0.001  0.045  <0.001  <0.001  0.040  0.095  0.003  <0.001  0.270  0.077  <0.001  <0.001  0.209  0.626  0.249  0.006  0.002  <0.001  –  <0.001  <0.001  0.137  0.024  0.005  0.167  0.052  0.014  <0.001  0.568  0.838  <0.001  <0.001  0.029  0.668  0.144  0.988  0.005  <0.001  0.214 |

All models were adjusted for vitamin D status, WC, age, gender, race/ethnicity, educational level, family income to poverty ratio, leisure-time physical activity, smoking and drinking.

* P<0.05

Abbreviations: HR, hazard ratio; CI, confidence interval; CVD, cardiovascular disease; BMI, body mass index.

**Supplementary Table 3.** The HRs (95% CIs) for all covariates for all-cause and cause-specific mortality in NHANES III and NHANES 2001–2014: model including vitamin D status and WC-categories

|  | WC | |
| --- | --- | --- |
|  | HR (95% CI) | P values |
| **All-cause mortality**  Age  Gender  Male  Female  Race  Non-Hispanic white  Non-Hispanic black  Mexican American  Other  Education  Less than high school  High school or equivalent  College or above  Family income-poverty ratio  ≤1.0  1.0 - 3.0  >3.0  Leisure-time physical activity  Active  Moderately active  Inactive  Smoking  Never  Former  Current  Alcohol  <14 g/d  14-28 g/d  ≥28 g/d  WC  Non-abdominal obesity  Abdominal obesity  Vitamin D status  Sufficiency  Insufficiency  Deficiency  Possibly harmful  **CVD mortality**  Age  Gender  Male  Female  Race  Non-Hispanic white  Non-Hispanic black  Mexican American  Other  Education  Less than high school  High school or equivalent  College or above  Family income-poverty ratio  ≤1.0  1.0 - 3.0  >3.0  Leisure-time physical activity  Active  Moderately active  Inactive  Smoking  Never  Former  Current  Alcohol  <14 g/d  14-28 g/d  ≥28 g/d  WC  Non-abdominal obesity  Abdominal obesity  Vitamin D status  Sufficiency  Insufficiency  Deficiency  Possibly harmful  **Cancer mortality**  Age  Gender  Male  Female  Race  Non-Hispanic white  Non-Hispanic black  Mexican American  Other  Education  Less than high school  High school or equivalent  College or above  Family income-poverty ratio  ≤1.0  1.0 - 3.0  >3.0  Leisure-time physical activity  Active  Moderately active  Inactive  Smoking  Never  Former  Current  Alcohol  <14 g/d  14-28 g/d  ≥28 g/d  WC  Non-abdominal obesity  Abdominal obesity  Vitamin D status  Sufficiency  Insufficiency  Deficiency  Possibly harmful | 1.08 (1.08 – 1.09) *  Reference  0.67 (0.63 – 0.70) *  Reference  0.95 (0.89 – 1.02)  0.82 (0.77 – 0.88) *  0.62 (0.55 – 0.71) *  Reference  0.84 (0.79 – 0.89) *  0.78 (0.73 – 0.84) *  Reference  0.87 (0.82 – 0.93) *  0.61 (0.56 – 0.65) *  Reference  0.93 (0.87 – 1.01)  1.07 (0.99 – 1.15)  Reference  1.25 (1.17 – 1.32) *  1.85 (1.73 – 1.97) *  Reference  0.91 (0.82 – 1.02)  0.91 (1.01 – 1.17) *  Reference  1.07 (1.02 – 1.13) *  Reference  1.17 (1.11 – 1.24) *  1.47 (1.35 – 1.61) *  1.00 (0.65 – 1.55)  1.10 (1.09 – 1.10) *  Reference  0.49 (0.44 – 0.56) *  Reference  0.87 (0.75 – 1.02)  0.70 (0.60 – 0.82) *  0.57 (0.42 – 0.77)  Reference  0.86 (0.75 – 0.99) *  0.88 (0.75 – 1.03)  Reference  0.82 (0.72 – 0.95) *  0.52 (0.44 – 0.61) *  Reference  0.90 (0.76 – 1.08)  1.15 (0.99 – 1.34)  Reference  1.30 (1.14 – 1.49) *  1.92 (1.66 – 2.23) *  Reference  0.85 (0.65 – 1.10)  0.96 (0.80 – 1.15)  Reference  1.26 (1.12 – 1.42) *  Reference  1.23 (1.08 – 1.39) *  1.62 (1.33 – 1.97) *  –  1.07 (1.07– 1.08) *  Reference  0.72 (0.65 – 0.81) *  Reference  1.10 (0.97 – 1.26) *  0.84 (0.73 – 0.97) *  0.69 (0.54 – 0.89) *  Reference  0.92 (0.81 – 1.04)  0.87 (0.76 – 1.00)  Reference  0.86 (0.76 – 0.97) *  0.71 (0.61 – 0.82) *  Reference  0.96 (0.82 – 1.11)  0.99 (0.86 – 1.14)  Reference  1.48 (1.30 – 1.67) *  2.40 (2.11 – 2.73) *  Reference  0.77 (0.61 – 0.98) *  1.03 (0.89 – 1.21)  Reference  0.96 (0.86 – 1.07)  Reference  1.18 (1.05 – 1.32) *  1.43 (1.20 – 1.70) *  1.55 (0.80 – 3.00) | <0.001  <0.001  0.136  <0.001  <0.001  <0.001  <0.001  <0.001  <0.001  0.087  0.068  <0.001  <0.001  0.107  0.034  0.010  <0.001  <0.001  0.982  <0.001  <0.001  0.081  <0.001  <0.001  0.037  0.104  0.004  <0.001  0.253  0.075  <0.001  <0.001  0.210  0.621  <0.001  0.001  <0.001  –  <0.001  <0.001  0.142  0.016  0.004  0.165  0.052  0.013  <0.001  0.574  0.890  <0.001  <0.001  0.030  0.661  0.441  0.004  <0.001  0.192 |

All models were adjusted for vitamin D status, WC, age, gender, race/ethnicity, educational level, family income to poverty ratio, leisure-time physical activity, smoking and drinking.

* P<0.05

Abbreviations: HR, hazard ratio; CI, confidence interval; CVD, cardiovascular disease; WC, waist circumference.

**Supplementary Table 4.** Supplementary models for the associations of serum vitamin D status and obesity levels with all-cause and cause-specific mortality in NHANES III and NHANES 2001–2014: HRs (95% CIs) for supplementary models across different BMI/WC categories

|  | Sufficiency | Insufficiency | | Deficiency | Possibly harmful | | |
| --- | --- | --- | --- | --- | --- | --- | --- |
| **All-cause mortality**  Supplementary Model 1  BMI  Normal weight  Overweight  Obese  WC  Not abdominally obese  Abdominally obese  Supplementary Model 2  BMI  Normal weight  Overweight  Obese  WC  Not abdominally obese  Abdominally obese  Supplementary Model 3  BMI  Normal weight  Overweight  Obese  WC  Not abdominally obese  Abdominally obese  Supplementary Model 4  BMI  Normal weight  Overweight  Obese  WC  Not abdominally obese  Abdominally obese  **CVD mortality**  Supplementary Model 1  BMI  Normal weight  Overweight  Obese  WC  Not abdominally obese  Abdominally obese  Supplementary Model 2  BMI  Normal weight  Overweight  Obese  WC  Not abdominally obese  Abdominally obese  Supplementary Model 3  BMI  Normal weight  Overweight  Obese  WC  Not abdominally obese  Abdominally obese  Supplementary Model 4  BMI  Normal weight  Overweight  Obese  WC  Not abdominally obese  Abdominally obese  **Cancer mortality**  Supplementary Model 1  BMI  Normal weight  Overweight  Obese  WC  Not abdominally obese  Abdominally obese  Supplementary Model 2  BMI  Normal weight  Overweight  Obese  WC  Not abdominally obese  Abdominally obese  Supplementary Model 3  BMI  Normal weight  Overweight  Obese  WC  Not abdominally obese  Abdominally obese  Supplementary Model 4  BMI  Normal weight  Overweight  Obese  WC  Not abdominally obese  Abdominally obese | HRs  1  1  1  1  1  1  1  1  1  1  1  1  1  1  1  1  1  1  1  1  1  1  1  1  1  1  1  1  1  1  1  1  1  1  1  1  1  1  1  1  1  1  1  1  1  1  1  1  1  1  1  1  1  1  1  1  1  1  1  1 | HRs  1.22 (1.09 – 1.36) *  1.17 (1.06 – 1.28) *  1.15 (1.04 – 1.28) *  1.18 (1.07 – 1.30) *  1.23 (1.09 – 1.39) *  1.20 (1.07 – 1.33) *  1.16 (1.05 – 1.27) *  1.15 (1.04 – 1.27) *  1.21 (1.11 – 1.32) *  1.13 (1.05 – 1.22) *  1.20 (1.07 – 1.33) *  1.17 (1.07 – 1.29) *  1.16 (1.05 – 1.28) *  1.21 (1.11 – 1.32) *  1.14 (1.06 – 1.23) *  1.18 (1.06 – 1.32) *  1.18 (1.07 – 1.30) *  1.16 (1.06 – 1.29) *  1.21 (1.11 – 1.32) *  1.15 (1.07 – 1.24) *  1.55 (1.20 – 1.99) *  1.11 (0.89 – 1.38)  1.27 (1.02 – 1.58) *  1.38 (1.12 – 1.71) *  1.21 (1.02 – 1.42) *  1.52 (1.19 – 1.95) *  1.08 (0.87 – 1.35)  1.27 (1.02 – 1.57) *    1.36 (1.11 – 1.68) *  1.21 (1.02 – 1.42) *  1.43 (1.12 – 1.82) *  1.08 (0.87 – 1.34)  1.26 (1.02 – 1.56) *  1.31 (1.07 – 1.61) *  1.18 (1.00 – 1.38) *  1.41 (1.10 – 1.81) *  1.06 (0.85 – 1.32)  1.25 (1.00 – 1.55) *  1.31 (1.06 – 1.61) *  1.17 (0.99 – 1.37)  1.21 (0.97 – 1.50)  1.17 (0.97 – 1.42)  1.19 (0.97 – 1.47)  1.21 (1.02 – 1.44) *  1.16 (0.99 – 1.36)  1.18 (0.95 – 1.47)  1.15 (0.95 – 1.40)  1.19 (0.97 – 1.46)  1.19 (1.00 – 1.42) *  1.15 (0.98 – 1.34)  1.17 (0.95 – 1.45)  1.17 (0.97 – 1.41)  1.19 (0.98 – 1.46)  1.20 (1.01 – 1.42) *  1.15 (0.99 – 1.34)  1.16 (0.94 – 1.44)  1.19 (0.98 – 1.43)  1.21 (0.99 – 1.48)  1.20 (1.01 – 1.42) *  1.17 (1.00 – 1.36) * | P  <0.001  0.001  0.005  <0.001  0.001  0.001  0.002  0.007  <0.001  0.001  0.001  0.001  0.003  <0.001  0.001  0.002  0.001  0.002  <0.001  <0.001  0.001  0.360  0.035  0.003  0.026  0.001  0.465  0.033  0.003  0.025  0.004  0.498  0.032  0.009  0.049  0.006  0.600  0.046  0.011  0.061  0.091  0.107  0.092  0.030  0.068  0.129  0.139  0.092  0.047  0.079  0.146  0.101  0.083  0.033  0.071  0.174  0.080  0.060  0.038  0.049 | HRs  1.57 (1.33 – 1.85) *  1.15 (1.04 – 1.28) *  1.46 (1.26 – 1.69) *  1.59 (1.38 – 1.83) *  1.44 (1.28 – 1.61) *  1.49 (1.27 – 1.76) *  1.41 (1.21 – 1.65) *  1.42 (1.23 – 1.64) *  1.52 (1.32 – 1.74) *  1.41 (1.26 – 1.58) *  1.50 (1.28 – 1.75) *  1.42 (1.21 – 1.66) *  1.43 (1.24 – 1.65) *  1.53 (1.33 – 1.75) *  1.42 (1.27 – 1.59) *  1.49(1.26 – 1.74) *  1.44 (1.23 – 1.68) *  1.42 (1.23 – 1.65) *  1.51 (1.31 – 1.73) *  1.43 (1.28 – 1.60) *  1.73 (1.17 – 2.56) *  1.80 (1.27 – 2.56) *  1.68 (1.21 – 2.33) *  1.93 (1.39 – 2.69) *  1.57 (1.21 – 2.03) *  1.67 (1.14 – 2.45) *  1.73 (1.23 – 2.44) *  1.64 (1.19 – 2.27) *  1.87 (1.35 – 2.57) *  1.54 (1.20 – 1.99) *  1.57 (1.08 – 2.29) *  1.70 (1.21 – 2.40) *  1.65 (1.20 – 2.28) *  1.80 (1.31 – 2.47) *  1.51 (1.18 – 1.95) *  1.57 (1.07 – 2.30) *  1.67 (1.18 – 2.35) *  1.61 (1.17 – 2.23) *  1.61 (1.32 – 2.50) *  1.47 (1.14 – 1.90) *  1.45 (1.05 – 2.00) *  1.61 (1.18 – 2.20) *  1.30 (0.94 – 1.80)  1.43 (1.09 – 1.89) *  1.42 (1.12 – 1.81) *  1.39 (1.01 – 1.90) *  1.56 (1.15 – 2.122) *  1.29 (0.94 – 1.76)  1.38 (1.05 – 1.81) *  1.40 (1.11 – 1.77) *  1.41 (1.04 – 1.92) *  1.63 (1.20 – 2.19) *  1.26 (0.92 – 1.72)  1.41 (1.08 – 1.84) *  1.42 (1.12 – 1.79) *  1.42 (1.04 – 1.93) *  1.68 (1.24 – 2.27) *  1.20 (0.87 – 1.66)  1.44 (1.10 – 1.87) *  1.39 (1.10 – 1.76) * | P  <0.001  <0.001  <0.001  <0.001  <0.001  <0.001  <0.001  <0.001  <0.001  <0.001  <0.001  <0.001  <0.001  0.001  <0.001  <0.001  <0.001  <0.001  <0.001  <0.001  0.006  0.001  0.002  0.018  0.012  0.009  0.002  0.003  <0.001  0.001  0.018  0.002  0.002  <0.001  0.001  0.020  0.004  0.004  <0.001  0.003  0.024  0.003  0.107  0.010  0.004  0.044  0.004  0.112  0.002  0.005  0.029  0.001  0.144  0.011  <0.001  0.028  0.001  0.258  0.008  0.006 | HRs  0.82 (0.39 – 1.72)  1.21 (0.57 – 2.54)  1.25 (0.56 – 2.79)  0.90 (0.46 – 1.73)  1.16 (0.65 – 2.05)  0.78 (0.37 – 1.65)  1.16 (0.55 – 2.44)  1.28 (0.57 – 2.87)  0.88 (0.46 – 1.69)  1.13 (0.64 – 2.00)  0.78 (0.37 – 1.65)  1.13 (0.54 – 2.39)  1.29 (0.58 – 2.88)  1.29 (0.45 – 1.67)  1.12 (0.64 – 1.99)  0.77 (0.37 – 1.63)  1.17 (0.56 – 2.48)  1.28 (0.57 – 2.87)  0.87 (0.45 – 1.67)  1.14 (0.65 – 2.02)  –  –  –  –  –  –  –  –  –  –  –  –  –  –  –  –  –  –  –  –  1.24 (0.40 – 3.91)  1.25 (0.31 – 5.03)  3.22 (1.19 – 8.73) *   - 1. (0.34 – 3.34)   2. (0.34 – 4.83)   1.18 (0.37 – 3.69)  1.19 (0.37– 4.80)  3.22 (1.19 – 8.72) *  1.02 (0.33 – 3.19)  2.10 (0.93 – 4.72)  1.21 (0.38 – 3.79)  1.21 (0.30 – 4.90)  3.19 (1.18 – 8.64) *  1.03 (0.33 – 3.23)   - 1. (0.93 – 4.69)   1.17 (0.94 – 3.68)  1.23 (0.33 – 4.96)  3.24 (1.20 – 8.76) *  1.03 (0.33 – 3.23)  2.12 (0.94 – 4.78) | P  0.595  0.623  0.593  0.741  0.616  0.522  0.702  0.546  0.699  0.676  0.520  0.745  0.538  0.668  0.688  0.501  0.672  0.545  0.673  0.650  –  –  –  –  –  –  –  –  –  –  –  –  –  –  –  –  –  –  –  –  0.709  0.758  0.021  0.909  0.065  0.781  0.808  0.021  0.973  0.074  0.749  0.785  0.022  0.956  0.076  0.784  0.771  0.021  0.956  0.069 |

Model 1: further adjusted for dietary supplement use (Yes or No), polyunsaturated fatty acids intake, calcium and magnesium intake.

Model 2: further adjusted for HEI scores.

Model 3: further adjusted for vegetables, fruits and grain HEI scores.

Model 4: further adjusted for GFR ≥ 90 mL/min per 1.73 m^2^ (Yes or No).

* P<0.05

Abbreviations: HR, hazard ratio; CI, confidence interval; CVD, cardiovascular disease; WC, waist circumference; BMI, body mass index; HEI, healthy eating index; GFR, glomerular filtration rate.

**Supplementary Table 5.** Baseline characteristics of participants included or excluded from analyses due to missing any of covariates, and comparison between them

| Characteristics | Included  N = 34172 | Excluded  N = 5886 | Total excluded  N = 11408 |
| --- | --- | --- | --- |
| Mean age in years ^a, b^  Gender  Male  Female  Race/ethnicity ^a, b^  Non-Hispanic white  Non-Hispanic black  Mexican American  Other  Education ^a, b^  Less than high school  High school or equivalent  College or above  Family income-poverty ratio ^a, b^  ≤1.0  1.0 - 3.0  >3.0  Leisure-time physical activity ^a, b^  Inactive  Moderately active  Active  Smoking ^a, b^  Never  Former  Current  Alcohol, g/d ^a, b^  <14  14-28  ≥28  BMI, kg/m^2 a, b^  <18.5  18.5 – 24.9  25.0 – 29.9  ≥30  Waist circumference ^b^  Not abdominally obese  Abdominally obese | 46.4 (46.3 - 46.6)  16658 (48.8)  17514 (51.3)  15421 (45.1)  7964 (23.3)  7022 (20.6)  3765 (11.0)  10607 (31.0)  10320 (30.2)  13245 (38.8)  7930 (23.2)  13758 (40.3)  12484 (36.5)  15973 (46.7)  11537 (33.8)  6662 (19.5)  17769 (52.0)  8341 (24.4)  8062 (23.6)  27662 (81.0)  2390 (7.0)  4120 (12.1)  544 (1.6)  10760 (31.5)  11681 (34.2)  11187 (32.7)  17014 (49.8)  17158 (50.2) | 49.7 (49.3 - 50.1)  2928 (49.8)  2958 (50.3)  2349 (39.9)  1291 (21.9)  1504 (25.6)  742 (12.6)  3003 (51.2)  1748 (29.8)  1120 (19.1)  986 (23.6)  1949 (46.7)  1237 (29.7)  999 (51.8)  647 (33.5)  283 (14.7)  2861 (48.7)  1417 (24.1)  1595 (27.2)  4497 (83.3)  355 (6.6)  550 (10.2)  104 (1.8)  1549 (26.3)  2050 (34.8)  2183 (37.1)  2497 (42.4)  3389 (57.6) | 49.5 (49.2 - 49.8)  5565 (48.8)  5843 (51.1)  4581 (40.2)  3024 (26.5)  2611 (22.9)  1192 (10.5)  4847 (42.9)  3636 (32.2)  2820 (25.0)  2175 (24.3)  4064 (45.4)  2720 (30.4)  3818 (54.6)  1951 (27.9)  1226 (17.5)  5528 (48.6)  2759 (24.2)  3095 (27.2)  7604 (83.6)  577 (6.3)  914 (10.1)  199 (2.1)  2707 (28.5)  3171 (33.3)  3438 (36.1)  3654 (44.6)  4543 (55.4) |

Data were N (%) or mean (95%CI).

Details of missing covariates among the total excluded: age (0), gender (0), race (0), education (105), family income-poverty ratio (2449), leisure-time physical activity (4413), smoking (26), alcohol (2313)

a P<0.05 (between included and excluded participants)

b P<0.05 (between included and total excluded participants)

P values were calculated using Mann-Whitney U test and χ2 test for continuous and categorical variables, respectively.

Abbreviation: BMI, body mass index.

**Supplementary Table 6.** The interaction effect of serum vitamin D status and obesity levels on all-cause and cause-specific mortality in NHANES III and NHANES 2001–2014: HRs (95% CIs) across different vitamin D status and obesity sub-categories: excluded participants with missing any of covariates

|  | Vitamin D sufficiency  HR (95% CI) | Vitamin D insufficiency  HR (95% CI) | Vitamin D deficiency  HR (95% CI) | Possibly harmful  HR (95% CI) |
| --- | --- | --- | --- | --- |
| **All-cause mortality**  Normal weight  Overweight  Obesity  Non-Abdominal obesity  Abdominal obesity  **CVD mortality**  Normal weight  Overweight  Obesity  Non-Abdominal obesity  Abdominal obesity  **Cancer mortality**  Normal weight  Overweight  Obesity  Non-Abdominal obesity  Abdominal obesity | Reference  0.92 (0.85 – 1.00)  1.08 (0.99 – 1.18)  Reference  1.12 (1.04 – 1.20) *  Reference  1.01 (0.84 – 1.23)  1.28 (1.05 – 1.58) *  Reference  1.34 (1.14 – 1.57) *  Reference  0.92 (0.78 – 1.09)  1.00 (0.83 – 1.21)  Reference  0.97 (0.84 – 1.13) | 1.20 (1.08 – 1.34) *  1.08 (0.98 – 1.20)  1.26 (1.14 – 1.39) *  1.25 (1.14 – 1.36) *  1.27 (1.17 – 1.38) *  1.39 (1.09 – 1.78) *  1.11 (0.88 – 1.40)  1.67 (1.34 – 2.08) *  1.35 (1.09 – 1.66) *  1.60 (1.33 – 1.92) *  1.27 (1.03 – 1.57) *  1.05 (0.86 – 1.29)  1.17 (0.95 – 1.43)  1.29 (1.08 – 1.53) *  1.07 (0.90 – 1.26) | 1.53 (1.30 – 1.80) *  1.30 (1.11 – 1.54) *  1.46 (1.25 – 1.70) *  1.56 (1.35 – 1.80) *  1.52 (1.34 – 1.72) *  1.69 (1.16 – 2.46) *  1.72 (1.20 – 2.45) *  1.90 (1.35 – 2.67) *  1.90 (1.38 – 2.62) *  1.86 (1.41 – 2.47) *  1.64 (1.20 – 2.24) *  1.46 (1.07 – 2.01) *  1.15 (0.83 – 1.61)  1.49 (1.13 – 1.96) *  1.35 (1.05 – 1.74) * | 0.64 (0.27 – 1.54)  1.05 (0.47 – 2.34)  1.09 (0.45 – 2.64)  0.83 (0.39 – 1.74)  1.06 (0.57 – 1.98)  –  –  –  –  –  0.88 (0.22 – 3.56)  1.29 (0.32 – 5.19)  2.41 (0.77 – 7.54)  0.78 (0.19 – 3.15)  1.78 (0.73 – 4.32) |

All models were adjusted for age, gender, race/ethnicity, educational level, family income to poverty ratio, leisure-time physical activity, smoking and drinking.

* P<0.05

Abbreviations: HR, hazard ratio; CI, confidence interval; CVD, cardiovascular disease.

**Supplementary Table 7.** Stratified HRs (95% CIs) across different BMI categories and additive effect of serum 25(OH)D status on all-cause and cause-specific mortality in NHANES III and NHANES 2001–2014: excluded participants with missing any of covariates

| Vitamin D status | BMI  Hazard ratio (95% CIs) | | | | | | | | |
| --- | --- | --- | --- | --- | --- | --- | --- | --- | --- |
|  | Normal weight | | | Overweight | | | Obese | | |
| **All-cause mortality**  Number of deaths  Sufficiency  Insufficiency  Deficiency  Possibly harmful  **CVD mortality**  Number of deaths  Sufficiency  Insufficiency  Deficiency  Possibly harmful  **Cancer mortality**  Number of deaths  Sufficiency  Insufficiency  Deficiency  Possibly harmful | N  1024  517  180  5  185  105  33  0  254  136  50  2 | 1726  1 (Reference)  1.21 (1.08 – 1.35) *  1.52 (1.28– 1.80) *  0.62 (0.25 – 1.49)  323  1 (Reference)  1.49 (1.15– 1.92) *  1.78 (1.20– 2.64) *  –  442  1 (Reference)  1.17 (0.94– 1.47)  1.44 (1.04– 1.99) *  0.82 (0.20 – 3.30) | P  0.001  0.001  0.280  0.003  0.004  0.166  0.030  0.775 | N  1252  671  174  6  264  123  39  0  301  158  49  2 | 2103  1 (Reference)  1.16 (1.05– 1.28) *  1.39 (1.18 – 1.65) *  1.12 (0.50 – 2.50)  426  1 (Reference)  1.07 (0.85– 1.34)  1.64 (1.14 – 2.36) *  –  510  1 (Reference)  1.15 (0.94– 1.41)  1.65 (1.19– 2.29) *  1.37 (0.34 – 5.54) | P  0.003  <0.001  0.787  0573  0.010  0.182  0.003  0.658 | N  895  701  218  5  199  160  46  0  201  157  43  3 | 1819  1 (Reference)  1.16 (1.04– 1.29) *  1.32(1.13– 1.56) *  1.11 (0.46 – 2.69)  405  1 (Reference)  1.25 (1.00 – 1.57)  1.37 (0.96– 1.96)  –  404  1 (Reference)  1.21 (0.97– 1.51)  1.25 (0.87 – 1.79)  2.67 (0.85 – 8.40) | P  0.001  0.007  0.811  0.051  0.086  0.096  0.223  0.094 |

All models were adjusted for age, race, gender, educational level, ratio of family income to poverty, leisure-time physical activity, smoking and drinking.

* P<0.05

Abbreviations: HR, hazard ratio; CI, confidence interval; BMI, body mass index; CVD, cardiovascular disease.

**Supplementary Table 8.** Stratified HRs (95% CIs) across different WC categories and additive effect of serum 25(OH)D status on all-cause and cause-specific mortality in NHANES III and NHANES 2001–2014: excluded participants with missing any of covariates

| Vitamin D status | WC  Hazard ratio (95% CIs) | | | | | |
| --- | --- | --- | --- | --- | --- | --- |
|  | Not abdominally obese | | | Abdominally obese | | |
| **All-cause mortality**  Number of deaths  Sufficiency  Insufficiency  Deficiency  Possibly harmful  **CVD mortality**  Number of deaths  Sufficiency  Insufficiency  Deficiency  Possibly harmful  **Cancer mortality**  Number of deaths  Sufficiency  Insufficiency  Deficiency  Possibly harmful | N  1488  789  238  7  266  148  48  0  385  214  62  2 | 2522  1 (Reference)  1.25 (1.14– 1.37) *  1.55 (1.34– 1.80) *  0.74 (0.35 – 1.57)  462  1 (Reference)  1.40 (1.13– 1.74) *  2.00 (1.44– 2.79) *  –  663  1 (Reference)  1.25 (1.04– 1.49) *  1.41 (1.06– 1.88) *  0.72 (0.18 – 2.91) | P  <0.001  <0.001  0.438  0.002  <0.001  0.016  0.018  0.646 | N  1737  1138  351  10  389  248  70  0  389  245  83  5 | 3236  1 (Reference)  1.12 (1.04– 1.22) *  1.34 (1.18– 1.52) *  1.02 (0.55 – 1.91)  707  1 (Reference)  1.16 (0.97– 1.37)  1.32 (1.00– 1.74)  –  722  1 (Reference)  1.11 (0.94– 1.32)  1.42 (1.09– 1.84) *  1.96 (0.81 – 4.76) | P  0.004  <0.001  0.946  0.054  0.098  0.223  0.008  0.138 |

All models were adjusted for age, race, gender, educational level, ratio of family income to poverty, leisure-time physical activity, smoking and drinking.

* P<0.05

Abbreviations: HR, hazard ratio; CI, confidence interval; CVD, cardiovascular disease; WC, waist circumference.

***Supplementary figures***


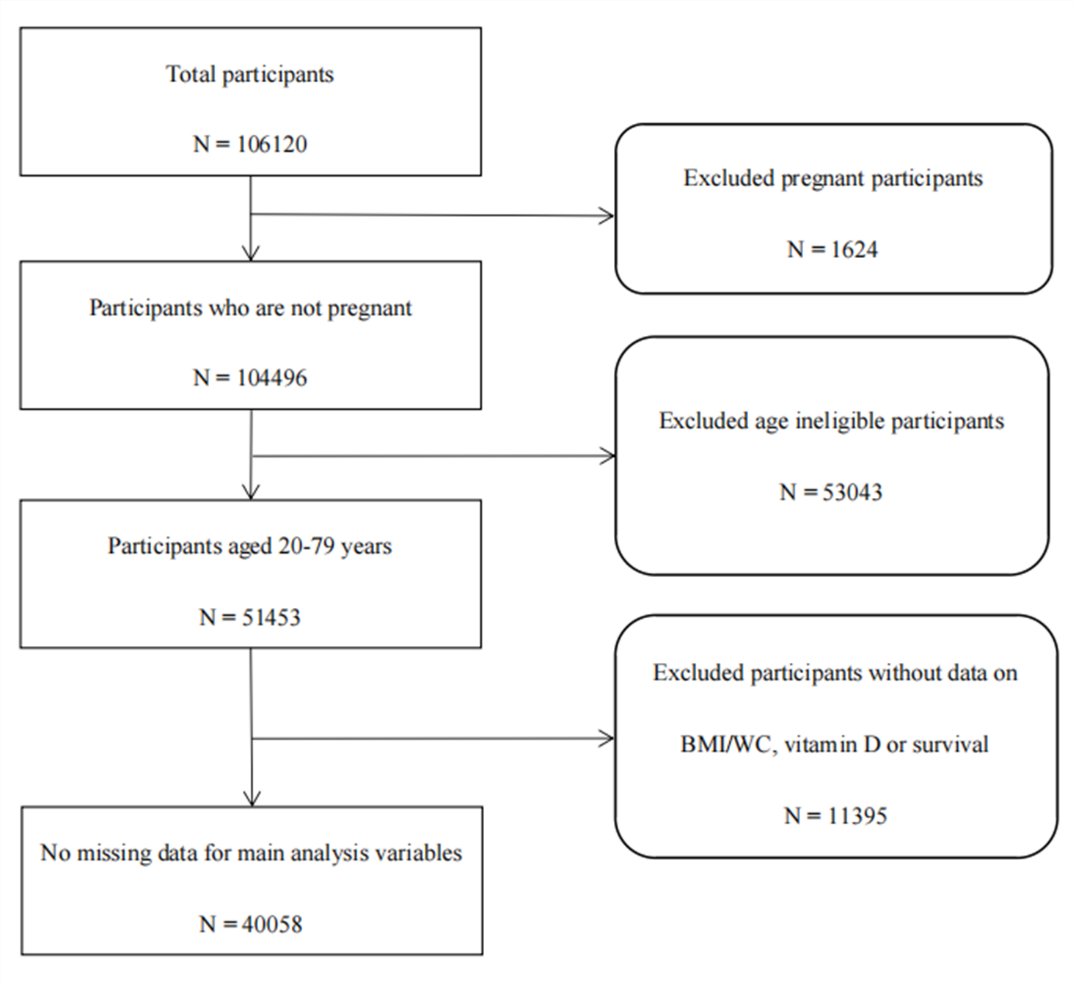


**Supplementary Figure 1.** Flow chart of study participants. Abbreviations: BMI, body mass index; WC, waist circumference.
